# Examining Feasibility, Acceptability, and Preliminary Outcomes of a Culturally Adapted Evidence-Based Postpartum Depression Preventive Intervention for Women in Doha, Qatar: Protocol for a Randomized Controlled Trial

**DOI:** 10.2196/11623

**Published:** 2023-08-11

**Authors:** Sawssan R Ahmed, Felice Watt, Ziyad Riyad Mahfoud, Mona Korayem, Sara Buhmaid, Medhat Alberry, Ibrahim Mamoun Ibrahim, S Darius Tandon

**Affiliations:** 1 Department of Psychology California State University-Fullerton Fullerton, CA United States; 2 Sidra Medicine Doha Qatar; 3 Weill Cornell Medicine-Qatar Doha Qatar; 4 Center for Community Health Northwestern University Feinberg School of Medicine Chicago, IL United States

**Keywords:** postpartum depression, intervention, prevention, cultural adaptation, Arabic

## Abstract

**Background:**

Postpartum depression and anxiety are the 2 most common perinatal mental health disorders, with prevalence rates higher among women living in the Middle East than in most Western countries. The negative outcomes associated with postpartum depression and anxiety are profound and include less responsive parenting and compromised infant and young child development. Although interventions exist to prevent postpartum depression and anxiety, to date, there have been no studies that have attempted to prevent postpartum depression or anxiety among Arabic-speaking women in the Middle East, including Qatar.

**Objective:**

The purpose of this study is to conduct a randomized controlled trial (RCT) of an evidence-based postpartum depression preventive intervention—Mothers and Babies (MB)—culturally adapted for use with Arabic-speaking women in Doha, Qatar. MB is guided by a cognitive behavioral therapy framework that focuses on increasing pleasant activities, promoting healthy thought patterns, and increasing the type and frequency of personal contacts. MB is tailored to specific needs and issues related to pregnancy and the postpartum period.

**Methods:**

A multidisciplinary and multiethnic investigator team adapted MB to promote cultural and contextual fit for Arabic-speaking women. Intervention concepts were reviewed to ensure core content would be understood by Arabic-speaking women in Qatar. Subsequently, images, activities, and examples in the intervention manuals were adapted, as needed, to make the content more relevant to Arab culture. To deliver the adapted intervention, 30 Arabic-speaking individuals with mental health backgrounds were trained. The adapted intervention was subsequently pilot-tested with 10 pregnant women receiving prenatal care at Sidra Hospital in Doha. We are now conducting an RCT to examine the effectiveness of the adapted intervention. We plan to enroll 210 pregnant individuals who are Arabic-speaking, with 1:1 randomization to the MB intervention or usual prenatal care. Among the enrolled participants, a stratified subsample of 40 pregnant women with diabetes is being recruited. Data collection will take place at baseline and a 6-month follow-up. The primary outcomes are depressive and anxiety symptoms and perceived stress. Diabetes self-care is the exploratory outcome for the subsample of individuals with diabetes. Intervention implementation will be assessed via client and provider surveys during and after intervention delivery. Power and sample size were calculated using a 2-sided 5% effort rate and assumed analyses on the individual level, accounting for attrition of 20%.

**Results:**

The cultural adaptation and pilot study of the adapted MB intervention are completed. A total of 157 women have been enrolled in the RCT as of March 31, 2023.

**Conclusions:**

This study is highly innovative, as it is the first study to our knowledge to examine the effectiveness of an evidence-based postpartum depression preventive intervention in the Middle East. Limitations include a single follow-up time point and a small subsample of individuals with diabetes.

**Trial Registration:**

ClinicalTrials.gov NCT04442529; https://www.clinicaltrials.gov/study/NCT04442529

**International Registered Report Identifier (IRRID):**

DERR1-10.2196/11623

## Introduction

Perinatal mental health (PMH) disorders are common and have significant impacts on women, their infants, families, and communities [1,2]. Postpartum depression and postpartum anxiety are the 2 most common PMH disorders [3,4]. Systematic reviews have found a pooled prevalence of 15.5% across studies conducted in high-income countries [5], while systematic reviews of studies conducted in low- and middle-income countries found a higher pooled prevalence of 24.7% [6]. Among low- and middle-income countries, the highest prevalence rates were found in the Middle East and North Africa. Studies conducted in Qatar have found postpartum depression prevalence rates ranging from 18.6% to 27.3% [7,8].

An array of efficacious interventions exist that prevent postpartum depression [9,10], with several of these interventions demonstrating effectiveness in improving anxiety symptoms given the comorbidity with postpartum depression [11]. One intervention that has demonstrated efficacy in reducing depressive and anxiety symptoms and preventing the onset of new cases of postpartum depression is the Mothers and Babies (MB) course [12-15]. MB is a manualized intervention guided by a cognitive behavioral therapy (CBT) framework. CBT is characterized by a focus on (1) increasing pleasant activities, (2) focusing on one’s thoughts, and (3) the type and frequency of personal contacts (ie, social support). MB contains a module in each of these 3 areas. The content of MB is tailored to specific needs and issues related to pregnancy and the postpartum period.

Despite the prevalence rate of postpartum depression exceeding those found in Western countries and the availability of interventions effective in preventing postpartum depression, to date, there have been no intervention studies that have attempted to prevent postpartum depression among Arab women in the Middle East and North Africa region, including Qatar. The overarching goal of this study is to examine the feasibility, acceptability, and outcomes associated with MB delivered to women in Doha, Qatar. However, because MB was developed and previously examined when delivered to Western pregnant women, cultural adaptation was required before rigorous testing of the intervention in Qatar. The specific aims of this study are:

1. To adapt MB for use with Arab Arabic-speaking women in Qatar. Cultural and contextual adaptations were undertaken by a multidisciplinary and multiethnic research team.
2. To conduct a small pilot study (n=10) that examines the acceptability, appropriateness, and feasibility of the adapted MB intervention when delivered to Arab Arabic-speaking women in Qatar. Pilot study findings are used to make final revisions to intervention and research protocols.
3. To conduct a randomized controlled trial (RCT; n=210) to determine the effectiveness of the adapted MB intervention on depressive symptoms, anxiety symptoms, and perceived stress among Arab Arabic-speaking women in Qatar. Our primary hypothesis is that women receiving the adapted MB intervention will exhibit greater reductions in depressive symptoms, anxiety symptoms, and perceived stress compared to women not receiving the intervention.

A subaim is to recruit a stratified random sample of 40 women with diabetes (n=20 control group; n=20 MB intervention) and examine intervention effects on mental health outcomes as well as diabetes self-management.

## Methods

### Ethics Approval

The study procedures described below have been approved by the institutional review boards of Sidra Medicine (2019-0004) and Northwestern University (STU00211149).

### Study Participants, Recruitment or Retention, and Informed Consent

A total of 210 women will be recruited. Women will be recruited from the prenatal care clinic at Sidra Medicine in Doha, Qatar. Women >18 years of age receiving prenatal care at Sidra Medicine—a women’s and children’s hospital—who are <28 weeks’ gestation at the time of study enrollment, are of Arab origin, and speak Arabic fluently will be eligible. Women meeting these criteria will be approached by Arabic-speaking research assistants while they are waiting for their prenatal health care appointment. Research assistants will administer the Edinburgh Postnatal Depression Scale (EPDS) [16] and the Generalized Anxiety Disorder-7 (GAD-7) item version [17]. To be eligible for the study, participants must score between 7 and 12 on the EPDS or 10-15 on the GAD-7, symptom ranges that are both indicative of mild to moderate symptoms of depression and anxiety, respectively. Participants who endorse a past history of major depression will also be eligible, while individuals endorsing current treatment for depression will be excluded given this study’s focus on the prevention of postpartum depression and anxiety. Other exclusion criteria have been minimized; however, women who self-reported receiving mental health treatment or having a serious mental health condition will also be excluded, as this may preclude women from regularly attending intervention sessions. Once eligibility is determined, the Arabic-speaking research assistant will introduce the study, answer any questions, and obtain signed informed consent from the participant.

Recruitment procedures emphasize the importance of participating in all MB sessions (for intervention participants) and remaining in the study through the 6-month follow-up assessment. The research team obtains ample tracking information at baseline, which includes the participant’s name, email address, home and cell phone numbers, and mailing address. The research team uploads contact information about each participant’s preferred mode of communication (eg, phone and text) at the time of recruitment.

### Randomization Procedures and Intervention Conditions

#### Randomization Procedures

Stratified permuted block randomization with blocks of sizes 2 and 4 will be used to randomize consenting women into the control arm or the intervention arm in a one-to-one fashion. The stratification will be based on the diabetic status of the woman. The randomization will be done by a statistician who is not involved in recruiting patients. A randomized list was created by the biostatistician, which was later uploaded to Research Electronic Data Capture (REDCap; Vanderbilt University) [18,19], a web-based software package for data management. This allows for the lack of human involvement on the part of the research team in the assignment of a participant to a group. It also allows for the on-the-spot assignment of participants into either the control or intervention groups.

#### Control Arm

Women randomized to the control arm will receive standard prenatal care delivered at Sidra Medicine.

#### Intervention Arm

Women randomized to the intervention arm will receive the culturally adapted MB intervention. MB consists of 12 sessions, each designed to last 15-20 minutes. The first 2 sessions provide (1) an understanding of the emotional and physical outcomes associated with stress, including concerns related to parent-child interaction, (2) strategies to notice one’s mood, and (3) an overview of how the remaining CBT content can help minimize stress levels. Sessions 3-5 focus on the CBT module of Pleasant Activities, while sessions 6-8 focus on Thoughts, and sessions 9-11 focus on Contact with Others. Session 12 is a course wrap-up and graduation. Intervention sessions provide concrete skills participants can use—for example, developing lists of pleasurable activities and strategizing how to overcome obstacles to engage in those activities. At the end of each session, a short personal project is provided to participants to encourage skill practice prior to the next session. Intervention sessions are delivered in-person, by phone, or via telehealth (Zoom and Microsoft Teams) by a mental health professional trained in MB. Sessions are intended to be delivered weekly or every other week, with flexibility given to deliver sessions at the same time to facilitate the timely completion of the intervention. Incentives will be provided to participants after completing each MB module to encourage their continued intervention participation. Additional content was added to selected sessions for the diabetic subgroup. This content was intended to draw links between the core MB cognitive-behavioral skills and diabetes management and self-care. For example, content that indicates how exercise and a healthy diet—aspects that are important for diabetes management—are also related to mental health, as both interventions have a positive effect on mental and emotional well-being, was added.

### Interventionist Training

Interventionist training in MB was provided by the senior author (SDT) and first author (SRA). Training was a 1-day in-person training conducted at Sidra Medicine. The first part of the training covered the conceptual underpinnings of MB (eg, its cognitive-behavioral framework), a brief history of previous implementation of MB, instruction on the format of the MB instructor manual, and discussion of implementation logistics. The second part of the training reviewed each of the 12 MB sessions from start to finish. Training was interactive, with opportunities for discussion and modeling the communication of material by the trainers. Training also involved group activities, where training attendees practiced delivering curriculum material and received feedback on strengths and areas needing improvement from the trainer and other training participants. We trained 30 individuals who met the following criteria: (1) employed as a nurse or health care professional or received graduate training in psychology; (2) were aged 18 years or older; and (3) had at least a bachelor’s degree in nursing or psychology, or equivalent mental health counseling training, experience, or certification. All trained individuals were fluent in Arabic.

### Data Collection Procedures and Study Assessments

The RCT includes 2 data collection time points—baseline and 6-month follow-up. We will attempt to collect 6-month assessments for intervention participants regardless of their engagement with the intervention. Participants will receive a remuneration of US $40 after completing each survey. Baseline and follow-up data will be collected and managed using REDCap. Women who do not choose to complete REDCap assessments will be contacted by phone by the research assistant to complete the assessment by phone or in person.

This study’s primary outcomes are a reduction in depressive symptoms, anxiety symptoms, and perceived stress. Secondary outcomes are closely linked with core MB content; secondary outcomes include behavioral activation, social support, mood regulation, decentering of thoughts, and MB skill use. The outcome associated with our exploratory aim is diabetes self-care. All measures have been validated in Arabic.

We will also assess various aspects of intervention implementation. We will conduct client surveys at the end of each MB module to assess clients’ perceptions of intervention acceptability and satisfaction. Each survey lasts 2-3 minutes and asks 3 questions pertaining to enjoyment with MB material, understanding of MB material, and anticipated use of MB material. Six months after a facilitator begins MB delivery with a client, the facilitator will receive a REDCap survey to report on the extent to which they covered all topics within an MB session (on average) and the extent to which clients were engaged in the MB material during intervention sessions.

### Safety and Data Monitoring

For any woman who endorses the suicidality question on the EPDS screener with a response that they think about suicide “quite often” or “sometimes,” the research assistant conducting the EPDS will escort the client to the outpatient mental health clinic, ensuring that the client is seen by a clinician. The clinicians in the Sidra Medicine outpatient clinic are trained to determine whether the client is in danger of harming herself or others and will determine the necessary steps to ensure the safety of the study participant. Regarding data monitoring, the study team will develop a series of data status and quality reports via an automated task, which study staff will review each week. These reports will focus primarily on reviewing missing data from collected assessments, accrual and retention, adverse events, and the number of women endorsing suicidal ideation.

### Statistical Analysis

A flowchart will be constructed based on the CONSORT (Consolidated Standards of Reporting Trials) guidance to show the number of recruited, excluded, lost to follow-up, and analyzed participants. Descriptive statistics will be used to summarize baseline characteristics overall and by study arm. As appropriate, mean (SD) and frequency (proportions) will be used to summarize continuous and categorical variables, respectively. Median (IQR) will be used in cases of skewed or nonnormal distributions. Analyses will use normal theory methodology as appropriate, and in cases of violations of assumptions, transformations or nonparametric analyses may be used. We will use SPSS (version 27; IBM Inc) to perform all analyses.

Analyses will be done using the intent-to-treat principle and will compare the 3 primary outcomes between study arms using the independent 2-tailed *t* test (or the Wilcoxon rank sum test in cases where outcomes were highly skewed). The analyses above will be repeated using univariate linear regression to be able to adjust later for any imbalances in baseline characteristics between the study arms and the baseline values of the primary outcomes. Additional analyses will include fitting multivariate linear regression models for each of the 3 main outcomes to assess the effectiveness of the intervention while adjusting for the baseline value of the outcome and any observed imbalances in participants between the 2 study arms. Unadjusted and adjusted mean differences in the primary outcomes between the 2 study arms will be presented along with their standard deviations and *P* values. For the primary outcomes, missing data at 6 months will be imputed using the average of the patients in that group or other methods such as multiple imputation by chained equations, and the primary analyses will be repeated as a sensitivity analysis. Secondary outcomes will be analyzed by comparing study arms using the chi-squared test (or Fisher exact test if expected cell counts are below 5) for categorical outcomes and the independent 2-tailed *t* test (or Wilcoxon rank sum test for highly skewed data) for numeric outcomes. Compliance with the intervention will be assessed by counting the number of intervention sessions received.

### Power and Sample Size Calculations

With 105 participants recruited per study arm and an estimated attrition of 20%, the study will be able to detect an effect size of 0.5 in any of the 3 primary outcomes using the independent 2-tailed *t* test with a power of 80% and a significance level of .017.

## Results

This study is in progress. We have completed the cultural adaptation of MB as well as the pilot study of the adapted MB intervention. Recruitment for the RCT began in February 2022.

### Cultural Adaptation of MB

A multidisciplinary Arabic-speaking research team comprised of psychologists, psychiatrists, obstetricians, endocrinologists, and nurses led the adaptation process. This research team was of different Arab backgrounds, with ethnic backgrounds represented including Qataris, Egyptians, Sudanese, Omani, and Palestinians. This combination of different ethnic backgrounds helped tailor MB to ensure a diversity of Arab cultures. The research team worked from the MB facilitator and participant manuals and reviewed them to first ensure that core intervention concepts would be understood by Arab-origin, Arabic-speaking women in Qatar. Additionally, images, activities, and examples found in the manuals were reviewed and adapted, as needed, to render the content more relevant and appropriate to Arab culture. For example, mindfulness sessions were adapted to include an Islamic and spiritual perspective to introduce the mindfulness sessions to the client (eg, how to improve the relationship with God, which can help in daily life and how to practice culturally informed mindfulness practices). Examples of pleasant activities that clients could engage in were modified to include culturally appropriate examples (eg, reading from the Quran and visiting family) and address contextual differences in activities (eg, removing examples of window shopping and gardening) that are not typically performed by Arab women in Qatar.

### Pilot Study of Adapted MB

A byproduct of this project is deepening the capacity of mental health professionals in Qatar to address mental health with their perinatal clients. As such, 29 individuals fluent in Arabic were trained on the adapted MB manuals. The individuals included graduate students from the Doha Institute for Graduate Studies’ new master’s in psychology program, medical students from Weill Cornell Medicine in Qatar, PMH nurses from Hamad Medical Corporation and Primary Health Care Cooperation, and nurses from Sidra Medicine. The nomination of these trainees was performed based on their medical backgrounds, prior experience with CBT, and availability to deliver the intervention during the time frame of the research study. Training was led by the 2 multiple principal investigators (SRA and SDT). Training occurred over a full day and introduced trainees to the MB intervention’s core concepts and implementation considerations. Subsequently, each intervention session was reviewed with key considerations highlighted by the trainers. Emphasis was placed on highlighting places in the curriculum where adaptations were made to promote cultural and contextual fit. A second training was held for 14 of the individuals who completed the first training and indicated an interest in facilitating MB intervention sessions. This second training provided these 14 individuals with details on the study’s intervention and research procedures (eg, facilitator responsibilities, patient recruitment process). Training was delivered in both Arabic and English.

For the pilot study, 4 of these facilitators delivered the MB intervention to 10 pregnant individuals. The average number of sessions received was 7.0 (out of 12), and 4 individuals completed all 12 sessions. Baseline assessments were conducted to pilot procedures and measures to be used in the RCT. Client and facilitator feedback was used to modify study procedures and slightly adapt the intervention content.

### RCT Recruitment

Recruitment for the RCT began in February 2022 and is expected to be completed by December 2023. As of March 31, 2023, a total of 259 pregnant women had been consented and screened for eligibility. Of those screened, 157 were eligible and enrolled in the study.

## Discussion

This study has several notable strengths and innovations. It is the first study to our knowledge that examines the effectiveness of a postpartum depression preventive intervention with Arab women in the Middle East, including Qatar. Given the higher prevalence of postpartum depression among women in the Middle East [6], interventions aimed at preventing and treating postpartum depression need to be examined in this context. The intervention tested in this study is an adapted version of an evidence-based postpartum depression preventive intervention—MB. Several RCTs of MB have demonstrated its effectiveness in preventing postpartum depression in diverse perinatal populations in the United States. However, given the significant cultural differences between the United States and Qatar, we engaged in a process of culturally adapting the intervention before testing its effectiveness with Arab-origin, Arabic-speaking women in Qatar. This cultural adaptation is another strength of this study, as a multidisciplinary team of researchers and clinicians from different Arab backgrounds engaged in an intensive process of reviewing and adapting the MB facilitator and participant manuals to ensure their cultural and contextual fit.

Our recruitment of a stratified subsample of diabetic women enhances the potential impact of this project. It is well established that stress during pregnancy has an impact on blood glucose control [20]. Pregnant women with diabetes who receive MB are likely to exhibit reduced stress levels by learning the cognitive, behavioral, and stress management techniques taught through the intervention. As a result, we anticipate that we will observe better diabetes self-management.

Because each MB session lasts only about 20 minutes, sessions can be delivered during an existing health care visit (eg, while waiting for a prenatal care appointment). MB is also highly flexible, allowing for intervention delivery in person, by phone, or via telehealth (eg, Zoom, Microsoft Teams). The use of non-face-to-face interventions may be of particular benefit to women who struggle to attend prenatal appointments—for example, if women do not feel well, have a child who is unwell, or have difficulty accessing transportation or childcare. The use of phone or telehealth approaches allows for the intervention to be more easily delivered during very hot weather or during Ramadan—important contextual considerations for interventions delivered to women in Qatar and the Middle East.

This study is not without limitations. Although the adaptation of MB was conducted to promote cultural and contextual fit, caution will need to be used in generalizing findings beyond the sample recruited from one medical center in Doha. Our 6-month follow-up time point is also limited in its ability to examine sustained intervention effects as well as the impact of the intervention on parenting or parent-child attachment outcomes. Although the diabetic subsample is innovative, our sample size is limited for this exploratory aim.

Pregnancy and the early postpartum period are times of increased risk of mental health disorders—particularly depression and anxiety—that impact not only maternal and fetal well-being and functioning but also mother-infant attachment, parenting practices, and infant development. These time periods are opportune times for mental health interventions given that women access health care more frequently and families are often more motivated to engage in health-promoting activities to promote positive outcomes for their children. This study has the potential to add to the evidence base on postpartum depression preventive interventions broadly and will also be the first of its kind to do so among exclusively Arab women in the Middle East.

## Appendix

Multimedia Appendix 1 Peer review report by the National Priorities Research Program, Qatar National Research Fund (Qatar).

## Abbreviations

CBT: cognitive behavioral therapy
CONSORT: Consolidated Standards of Reporting Trials
EPDS: Edinburgh Postnatal Depression Scale
GAD-7: Generalized Anxiety Disorder-7
MB: Mothers and Babies
PMH: perinatal mental health
RCT: randomized controlled trial
REDCap: Research Electronic Data Capture

## Data Availability

The data sets generated or analyzed during this study are available from the corresponding author upon reasonable request.
