# Supplementary material for: Examining Feasibility, Acceptability, and Preliminary Outcomes of a Culturally Adapted Evidence-Based Postpartum Depression Preventive Intervention for Women in Doha, Qatar: Protocol for a Randomized Controlled Trial
Source: JMIR Res Protoc. 2023 Aug 11;12:e11623. doi: 10.2196/11623 (PMC10457694; doi:10.2196/11623)
Supplement: Multimedia Appendix 1 [file resprot_v12i1e11623_app1.pdf]

QNRF Reviewer Comments

Panel Comments to LPI
NPRP11S-0121-180354
Qatar Mothers and Babies: examining the feasibility, acceptability, and preliminary outcomes associated with integrating a postpartum depression intervention into perinatal services for women.

Strengths
The study is aligned with Social Science, Arts and Humanities pertaining to 'Behaviors and interventions that improve quality of life and decrease risk'. The project has a significant training component addressing health personnel of different levels. Major health and teaching institutions are involved and the hiring of research assistants is foreseen. this would be the first instrument of this type available for arabic speaking women and could then be used also in other arabic speaking countries showing Qatar leadership in this area.

Weaknesses
The RCT is missing detailed protocol and also it is unclear why a sub-group of women with diabetes is included. Description of non-monetary incentives needs to be included to assess for coercion. The Stakeholder Advisor wondered if the study topic is priority to Qatar!

Conditions of the Award
To complete the RCT protocol.

Synthesis statement
The Mothers and Babies program has been identified as a viable and potentially impactful intervention to meet the needs of women exhibiting depressive and anxiety symptoms during the perinatal period. The reviewers supported the application since it is a very clear program based on established methodology, and that the intervention will provide a cost-effective, easy to implement approach to tackle postpartum depression. The study meets the current demands of mental health services in Qatar, hence the consensus was to support the application.

Taking into account all the proposals shortlisted in this NPRP-S cycle as well as the reviewers' comments and the panel discussion, the panel placed this proposal into the B category.

(Note: The eventual funding decision by QNRF will be subject to the availability of budgets.)

Reviewer - 1 Evaluation
Criterion: Intellectual Merit

Answer

N/A

Strengths

Perinatal mental health disorders have a substantial impact on the physical and psychological health and well-being of the mother, child and other family members. It is well established that experiencing depressive and anxiety symptoms during pregnancy are commonplace, yet little is known about prevalence and outcomes and specific risk factors in Middle Eastern women compared to Western women. This very well written and thought out proposal has several novelties. It will add to a sparse literature by obtaining prevalence data on perinatal affective disorders among Qatari women, risk factors specific to this group and will develop a well-established intervention to be culturally appropriate. For me, this is the major strength of this study, as other researchers have failed to adapt interventions to be contextually appropriate. (They have instead merely tried to translate the intervention). The higher prevalence of affective perinatal disorders reported in Qatari women requires further examination, including the phenomenology and clinical presentation. The data generated from this study will enable clinicians to gain understanding of the cultural presentation of perinatal mood disorders and compare to existing literature. The intervention will provide a cost-effective, easy to implement effective treatment for perinatal women that can be adapted relatively easily.

Weaknesses

It is unclear why a sub-group of women with diabetes is included. Although I draw my own rationale, the authors need to concisely present why this group has been included and how it adds to the literature and fits in with future studies/goals. It is unclear why the CES-D would be used as an outcome measure but included in baseline recruitment in conjunction with the EPDS.

Criterion: Expected Impact

Answer

N/A

Strengths

This is a very impressive multi-disciplinary research team. I had minor concerns regarding areas of expertise, however, the combination of investigators really does provide a complimentary and cohesive set of skills and experience. The early identification and treatment of affective disorders during the perinatal period can mitigate adverse outcomes that have health and financial outcomes. Infants born to mothers who experienced depression or anxiety during pregnancy are at risk of lower birth weight, altered immune function and lower cognitive scores and increased behavioral disturbances. These effects can persist into adolescence if untreated. Treatment of mothers will affective disorders has societal benefits - not only addressing infant outcomes (as listed above) but family functioning, work performance and longer term health outcomes including autoimmune disorders, recurrent depressive episodes and decreased parental interaction with children. Postpartum depression is the most common complication of childbirth. Decreasing stigma around these issues and increasing awareness is a huge public health concerns. Screening for these disorders is important, however providing effective treatment and access to services by mental health professionals has been a major challenge. This study would address this. The research team has a strong record of successfully completing funded studies, conferences presentations and publications in relevant peer reviewed publications. I am confident that they can successfully complete this study. Dr. Ahmed will also benefit in terms of professional development from being PI on a larger study under the supervision of Prof. Konje an Dr. Ibrahim. This is not to undermine her achievements to date but rather to develop management skills for a large grant and establish her for future studies and funding.

Weaknesses

The only part that is not well described is the potential impact of the diabetes sub-group analysis, and how this will be incorporated or developed.

Criterion: Work Plan

Answer
N/A
Strengths
The work plan is very clearly described with clear allocation of responsibilities and projected timelines. The fact that the team has a strong track record and the clinical team has established working relationships increases the likelihood of success. Dr. Tandon is very experienced in the training and implementation of the Mother Baby intervention and will be available during the in-person training in Qatar to share his experiences. The team has been carefully constructed to ensure that the project has the required expertise, reasonable time lines and the pilot study on 10 subjects will provide the opportunity to identify any problems before scaling up. Very thoughtful and I have no concerns.
Weaknesses
None identified.

Criterion: Qualifications and complementarity of the Research Team

Answer
N/A
Strengths
The team has been carefully put together and each brings a unique and complimentary skill to successfully complete the project. I am particularly pleased that Qatari women will have a prominent role as I think that this will significantly aid in the contextual development of the intervention. The PIs have a strong record of successful completion of research projects, timely dissemination of results at national and international conferences and peer reviewed publications. My understanding is that this will be the largest and most complex grant that Dr. Ahmed has led to date. However, her clinical and research experience is very impressive and she is poised to develop into an independent researcher of larger studies. This will provide an excellent opportunity for her and her professional growth. I would hope that the other senior team members (notably Prof. Konje, Dr. Ibrahim and Dr. Tandon) will mentor her and share their wisdom in running such studies.
Weaknesses
None identified.

Criterion: Budget

Answer
N/A
Strengths
The budget is appropriate with no concerns. The logistic and financial support from Sidra Medical (approximately $700k for salary support) shows the obvious support and clinical importance of this topic for the organization.
Weaknesses
None identified.

Criterion: Research Compliance

Answer
I have no major concerns for human subjects. Inclusion is appropriate and well presented. Two points that need clarification. As written, it appeared that Northwestern IRB application would happen first. As Sidra would have indemnity please reword so that it is clear that two separate applications will be submitted - Sidra and then Northwestern for de-identified data analysis only. If it's one application then specify who has indemnity and whether submissions would occur simultaneously. Regarding endorsement of suicidal ideation, '(women) will be offered' needs to changed to " (women) will be referred to same day review by Sidra women's mental health clinicians". A subtle difference but those endorsing suicidal ideation or intent to harm others must be assessed by a licensed mental health professional. The procedure for this needs to be strengthened as it is one of the potential harms. The fact that perinatal psychiatry experts are on the research team should be specified here, as well as their physical location in relation to where the subjects would be routinely seen. For example, mental health clinicians are located in the same building as OB services. or a clinician will be available to assess women and will be notified by telephone and

will come to the subjects location for assessment. It cannot be implied that the subject will be left to organize their assessment or could choose not to be assessed. What would happen if via Skype or Zoom women disclosed suicidal, homicidal or infanticidal thoughts? State the procedure (it could be calling emergency services but it needs to be specified). The non-monetary incentives are referred to on several occasions but are not specified. What are they?

Strengths

N/A

Weaknesses

N/A

Criterion: Overall Summary

Answer

This is a very well written, scientifically and clinically important study with an experienced and complimentary research team. It will directly benefit Qatari women, health organizations as well as provide novel data to the perinatal psychiatry field. The cultural adaptation of a well proven intervention to reduce postpartum depression symptoms is well thought out and of great importance. Minor issues are expounding upon the need for, and relevance of the sub-group of women with diabetes and firming up suicidal ideation screening. Description of non-monetary incentives needs to be included to assess for coercion. Similarly clarification on the IRB applications is required. Overall, a very strong and relevant application for a major public health concern.

Strengths

N/A

Weaknesses

N/A

Reviewer - 2 Evaluation

Criterion: Intellectual Merit

Answer

N/A

Strengths

The authors provide a very strong premise and intellectual merit for their proposal. Perinatal mental health (PMH) disorders have a substantial impact on society as the rates of depression and anxiety are known to be high in women of child bearing age. They provide evidence of high rates among Arab countries and point to recent findings of high rates of depression and anxiety among women living in Qatar. The objectives outlined in this proposal are clearly stated and achievable. This is essentially an implementation proposal and builds on existing evidence that an education and training program will provide care to women in the perinatal period and mitigate or prevent psychiatric disorders. The investigators have a strong plan to adapt the methods specifically to women living in Qatari society. The investigators demonstrate a high level of social awareness in working on adapting the methods specifically to Qatar. The instruments are available in Arabic, however they point out the need to adapt specifically to the social environment of Qatar.

Weaknesses

The approach is based on extant methods that are novel to Qatar, but are established in the western research environment.

Criterion: Expected Impact

Answer

N/A

Strengths

The potential impact for this program is very high. It addresses a profound need for mental health services that are efficient and designed with the culture and stressors that are specific to Qatar. The benefits are the establishment of a health educational program designed for Qatar mothers and babies with the goals of improving mental health of both. It uses an established method, adapted to Qatar and trains local health educators to engage local patients. It takes advantage of established

expertise to amplify the impact of the program. Once established the health educators will be able to train others to teach the program, thus expanding the program.

Weaknesses

This is a very clear program based on established methodology; no weakness at the level of impact are identified.

Criterion: Work Plan

Answer

N/A

Strengths

The work plan is very strong. Even though there is an extant Arabic version of the Mothers and Babies program instrument, it is expected that there will be elements that are unique to the culture and environment of Qatar that will be needed to be accounted for. Once this is in place the investigators will do a feasibility study to test the modified instrument, and make adaptations based on this study. They will then embark on a randomized clinical trial that is sufficiently powered to test the hypotheses that are proposed. The approach and plans are adaptive and accommodate to the preferences of the participants, e.g. whether the participants will receive the curriculum in person, by phone, or by telehealth media. They will use state of the art measures and instruments to gauge the outcomes of the participants.

Weaknesses

There are no weaknesses identified in this thorough work plan as outlined. The investigators are very organized.

Criterion: Qualifications and complementarity of the Research Team

Answer

N/A

Strengths

Very qualified and established team of investigators with a broad range of expertise. The LPI is well suited to coordinate the project and research teams.

Weaknesses

none identified

Criterion: Budget

Answer

N/A

Strengths

budget appears to be appropriate

Weaknesses

no weaknesses

Criterion: Research Compliance

Answer

All compliance matters are appropriate and well documented.

Strengths

N/A

Weaknesses

N/A

Criterion: Overall Summary

Answer

This is a well written and well organized proposal to adapt the Mothers and Babies program to Qatar. It begins with a adaptation of the extant program in Arabic to account for local cultural and environmental conditions (the concerns of women in this age group) in Qatar. It proceeds to a feasibility trial and then on to a randomized clinical trial that is sufficiently powered and uses standard statistical methods to evaluate outcomes. The potential impact is very high. The capacity building inherent in the program includes the ability of the trained professionals to continue educating others and thus amplifying the program. The attention to mother's health will benefit the society tremendously.

Strengths
N/A
Weaknesses
N/A

Reviewer - 3 Evaluation
Criterion: Intellectual Merit
Answer
N/A
Strengths
There is great societal need for an evidence-based program for perinatal depression that is adapted and usable for the local people. The original program appears to be a standard, well-established program already in wide use in USA, so developing and adapting its Arab version and validating it in an RCT is a standard and essential procedure.
Weaknesses
It is great that the authors have included the plan for RCT in their study project.
Criterion: Expected Impact
Answer
N/A
Strengths
The program, if validated in the Arab context, will offer a new standard for mothers and babies in Qatar.
Weaknesses
None.
Criterion: Work Plan
Answer
N/A
Strengths
Three-phase study is reasonable.
Weaknesses
The RCT is the essence of this whole research. However, the outline for this RCT is not up to the current scientific standard. 1) The primary outcome (including its measurement time) is not specified. No current trial registry would accept such a proposal. 2) The sample size calculation is wrong. You need only 30 per am to detect an effect size of 0.7 at alpha=0.05 and beta=0.20. So having 60 per arm allows you to detect an effect size of 0.5 at alpha=0.05 and beta=0.20, which is a good thing. On the other hand, among the subsample of diabetic mothers, 20 per am would allow you to detect an effect size almost as large as 1.0, which is quite unlikely. The researchers need to think about how to place this subsample within the larger trial. I would suggest that it be placed only as an exploratory subgroup analysis, without hypothesis testing. I would also like to argue for oversampling of diabetic mothers because it may be difficult and also because it may defeat the primary objective of the study. 3) CESD is not the current standard to measure depressive symptomatology in general and not for perinatal depression either. (CESD was originally developed to screen for depression, and I know many studies have used it but they are mainly old studies. You had better use a depression scale that is more sensitive to change.)
Criterion: Qualifications and complementarity of the Research Team
Answer
N/A
Strengths
It is great to have this international team, a part of which has developed the original intervention program.
Weaknesses
As has been reviewed in the Work Plan, the plan for the RCT is suboptimal. More expert input into the trial design is necessary.

Criterion: Budget

Answer
N/A
Strengths
Reasonable.
Weaknesses
None.

Criterion: Research Compliance

Answer
I do not see any ethical problem in the proposed plan.
Strengths
N/A
Weaknesses
N/A

Criterion: Overall Summary

Answer

As noted in Work Plan, the protocol for the RCT in Phase 3 needs polishing up. Please consult the methodological experts in the field. The original plan is reasonably (three phases to locally adapt the standard English program), and its societal and research impact will be great, if the RCT is properly conducted.

Strengths
N/A
Weaknesses
N/A
